# Major pathogenic *Clostridia* in human and progress toward the clostridial vaccines

**DOI:** 10.22038/IJBMS.2022.65518.14417

**Published:** 2022-09

**Authors:** Lida Abdolmohammadi Khiav, Azadeh Zahmatkesh

**Affiliations:** 1 Department of Anaerobic Vaccine Research and Production, Specialized Clostridia Research Laboratory, Razi Vaccine and Serum Research Institute, Agricultural Research, Education and Extension Organization, Karaj, Iran

**Keywords:** Botulinum, Clostridium, Difficile, Tetani, Toxin, Vaccine

## Abstract

The *Clostridium* genus is composed of a large spectrum of heterogeneous bacteria. They are Gram-positive, mostly mesophilic, and anaerobic spore-forming strains. *Clostridia* are widely distributed in oxygen-free habitats. They are found principally in the soil and intestines of ruminants as normal flora, but also are the cause of several infections in humans. The infections produced by important species in humans include botulism, tetanus, pseudomembranous colitis, antibiotics-associated diarrhea, and gas gangrene. Immunization with toxoid or bacterin-toxoid or genetically modified or other vaccines is a protective way against clostridial infection. Several experimental or commercial vaccines have been developed worldwide. Although conventional vaccines including toxoid vaccines are very important, the new generation of vaccines is an effective alternative to conventional vaccines. Recent advances have made it possible for new vaccines to increase immunogenicity. This review discusses briefly the important species of clostridia in humans, their toxins structure, and vaccine development and usage throughout the world.

## Introduction

**Figure 1:**
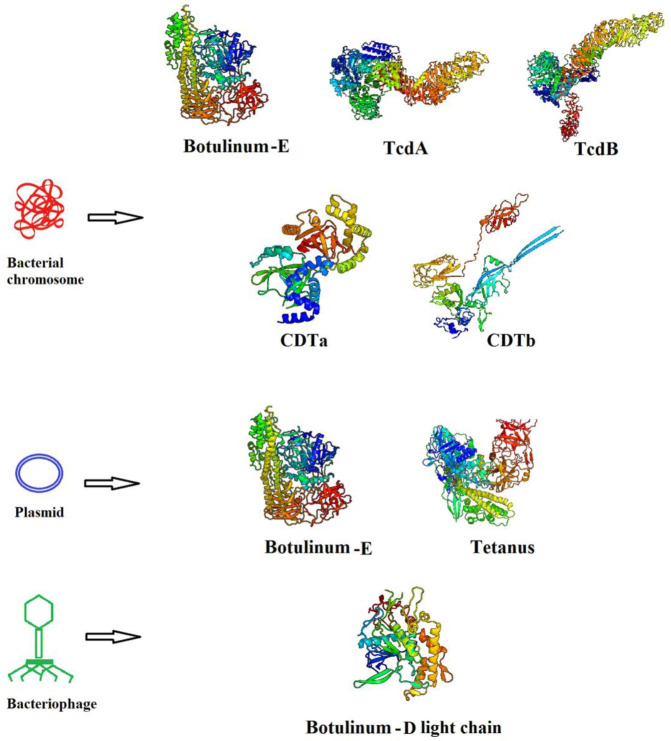
3D structure of human clostridial major toxins and their genetic origins. Botulinum toxin type D light chain was selected as a part of toxin structure

*Clostridia *genus is widespread worldwide and is commonly found in soil and animal and human intestines. They can survive in harsh environments for a long time due to their ability to form spores. Some organisms of this genus benefit agriculture by nitrogen fixation and phosphate solubilization and some of them are harmful to humans because of causing diseases such as tetanus, botulism, pseudomembranous colitis, food poisoning, and gas gangrene. Furthermore, some of them are the cause of serious diseases in animals due to their virulence factors (e.g., toxins) (1).* Clostridium* toxins are causative agents of mild to fatal diseases (2). *Clostridium botulinum* (*C. botulinum) *and *Clostridium tetani* (*C. tetani) *are the two causative agents of disease in man that produce two powerful toxins named (BoNT and TeNT), respectively. They belong to the *Firmicutes* phylum including approximately 231 species. They are anaerobic spore-forming, Gram-positive rods (3). This paper presents clostridia-causing diseases in humans and discusses the toxins, indicating the 3D structures of toxins (accessed by PHYRE2 server) (Figure 1) and their genetic origins as well as their mechanisms of action. Also, the experimental or commercial vaccines available in the world are described. 

***Clostridium botulinum***

*C. botulinum *is the etiological agent of a lethal paralytic disease named botulism. It is an obligate anaerobe, Gram-Positive, rod-shaped bacterium with oval, sub-terminal, and bulging spores.  *C. botulinum* is approximately 5 µm ×1.0 µm in dimensions and is arranged singly, in pairs, or short chains. *C. botulinum *is motile by peritrichous flagella. On sheep blood agar, it forms gray, smooth, translucent–opaque, and large (2-3 mm) colonies, with beta hemolysis. Mouse lethality bioassay (MLB) is the standard method for the detection of *C. botulinum *(4)*. *

*C*. botulinum is classified into four groups based on metabolic, physiological, and genetic aspects (5). Group I is composed of mesophilic and proteolytic bacteria, which can ferment some carbohydrates and digest casein or meat proteins. Group II is composed of psychrotrophic bacteria that can ferment some carbohydrates but cannot digest protein components. Group III is composed of proteolytic and saccharolytic bacteria. Today, group IV is located in a separate group from botulism cases (6). Gene encoding botulinum neurotoxins (BoNTs) are located on chromosome/plasmid in *C. botulinum* groups I and II, and bacteriophage and plasmid in *C. botulinum* groups III and IV, respectively. *C*. botulinum is classified into seven serogroups (A–G) based on the location of genes encoding BoNTs on chromosomes, plasmids, or phages (6). Group I is composed of A, B, E, and F, and group II is composed of types B, E, and F, and they both encompass human and animal cases, especially human botulism cases (7). Group III is composed of C and D and group IV is composed of type G (8). 

BoNT (molecular weight ~150 kDa) is a binary toxin including a heavy chain (HC) (~100 kDa) and a light chain (LC) (~50 kDa) connected by a disulfide bond (9). The toxin is activated when it is cleaved into the 100-kDa HC and the 50-kDa LC. Also, the HC is cleaved into two fragments of the N-terminal (translocation domain) and the C-terminal (receptor binding domain) domains. When the HC binds to the cholinergic nerve terminal, it enters the cells by endocytosis and results in LC activity. The N-terminal domain of the LC has metalloprotease activity. Consequently, it will cleave the components of SNARE (SNAP Receptor) proteins, causing blockade of acetylcholine release and flaccid muscle paralysis (10). 

*C. botulinum* can be differentiated from other clostridia on the basis of cultural and biochemical aspects and confirmed by the neutralization test (11). The Centers for Disease Control (CDC) has divided human botulism into five groups based on transmission route: foodborne, infant, wound, adult intestinal toxemia, and iatrogenic botulism (12).

Food-borne botulism occurs due to some processes such as home canning with anaerobic, low-acid, low-solute conditions. Wound botulism is a similar condition (anaerobic) observed in wounds. *C. botulinum* can germinate in the body and produce disease. Infant botulism was described in 1976 by Midura and Arnon and by Pickett *et al*. Honey and powdered infant milk are the main food sources of infant botulism. *C. botulinum* can germinate in the gut of humans because of low-acid conditions, decreased normal flora in the body, and immature immune systems (specifically lacking secretory immunoglobulin A). The symptoms of infant botulism are constipation, lethargy, a weak cry, breathing problems, poor feeding, and dehydration.

The botulinum neurotoxin is also considered the most potent toxin that can infect humans with a small dose (13). This toxin is listed as a bioterrorism agent (14). The consumption of botulinum toxin started at least 80 years ago. Although food-borne botulism has decreased in industrialized countries, it has not decreased in developing countries (15). If botulism is not treated, it can even lead to death due to difficulty in breathing, and muscle paralysis. Administration of polyvalent antitoxins reduces the clinical symptoms (16).

***Clostridium tetani***

C. tetani is the etiological agent of a fatal neuroparalytic disease called tetanus. C. tetani infects both humans and animals (17) with mortality throughout the world (18). *C. *tetani is an obligate anaerobic, Gram-positive, and rod-shaped bacterium with round, terminal, and bulging spores that appear with a drumstick shape under the optical microscope. *C.* tetani has approximately 4-8 µm ×0.5 µm dimensions arranged singly, in pairs, or short chains. C. tetani is motile by peritrichous flagella (19).  On sheep blood agar, it forms gray, rough colonies with alpha hemolysis, followed by beta hemolysis. C. tetani can be detected in culture supernatant by the mouse toxicity test and monitoring them for spastic paralysis. Also, C. tetani can be identified by ELISA and PCR-based methods (20). The origin of tetanus dates back to the 5th century BC (21) and it remains a concern in some countries, especially in non-industrialized countries (22). C. tetani spores germinate, grow, and release the tetanolysin and tetanospasmin (19). The gene encoding tetanolysin is located on the chromosome. Tetanolysin is an oxygen-sensitive hemolysin. It causes changes in the permeability of biological membranes resulting in tissue necrosis (23). Tetanus toxin, spasmogenic, or TeNT is an extremely potent toxin and the second known potent toxin in the world. 

The gene encoding tetanus-toxin is located on a 75-kb plasmid (23). Tetanus toxin (molecular weight ~150 kDa) is synthesized, as a polypeptide chain, which is cleaved by protease and produces a heavy chain (HC) (100 kD) and a light chain (LC) (50 kD) that are linked by a disulfide bond. The HC bonds gangliosides on neural tissues (24). The heavy chain is cleaved by the papain protease and produces two N-terminal (HN) and C-terminal (HC) fragments, the latter of which is responsible for binding to the target cell (25). The HN fragment participates in internalization, retrograde axonal transport, and translocation of the light chain into the neuronal cytosol. The light chain inhibits the release of inhibitory neurotransmitter glycine and gamma-aminobutyric acid at motor nerve endings. This act leads to spasming of muscles and even death (26). Other virulence factors related to tetanus disease include collagenase, hemolysin, and fibronectin-binding protein. The genes encoding collagenase, hemolysin, and fibronectin-binding protein are located on chromosomes (27). Fibronectin-binding protein causes colonization of bacteria in wounds and blood coagulation (28).

C. tetani spores enter the body through puncture wounds, lacerations, burns, or fractures (29). Also, the improper sterilization of surgical instruments or household knives, razor blades, or scissors used to cut the umbilical cord is the cause of neonatal tetanus(30). The main clinical symptoms of patients with neonatal tetanus are lack of sucking, trismus, spasticity, seizure, fever, cyanosis, omphalitis, and respiratory distress (31). In animals, it enters through castration, tailing, tagging, shear cuts, and fight bites (32). Horses are more susceptible than other animals, followed by small ruminants (33). Tetanus cases can be prevented by vaccination programs of the countries (34). Vaccinating pregnant women and girls before marriage can be effective. Also, increased number of rural and urban health centers, training of midwives and general practitioners, and enhancement of educational programs through media and vaccination programs have made great achievements (35).  In Iran, vaccination programs against diphtheria, tetanus, and pertussis (as a trivalent vaccine) have been implemented since the 1950s, but vaccination campaigns began in 1965. Today, a multivalent vaccine is administered in childhood, and after a 10-year period as a booster. 

***Clostridium*** ***difficile*** 

*Clostridioides difficile* infection (CDI or C-diff), also known as *C. difficile* is due to a spore-forming Gram-positive bacterium with rod shape and oval spore that arrange terminally or sub terminally, and are motile and capsulated. The colony appears on blood agar with slight irregularities of the edges, slightly raised, and semi-opaque to opaque. The bacterial arrangement is singly or in pairs and occasionally in short chains (36). 

*C. difficile* causes pseudomembranous colitis and antibiotics-associated diarrhea in humans. The three protein toxins of *C. difficile* are toxins A (TcdA), B (TcdB), and *C. difficile* transferase toxin (CDT). TcdA (308 kDa) (enterotoxin) and TcdB (270 kDa) (cytotoxin) are located on a chromosomal location called the pathogenicity locus (approximately 19.6-kb) and 1,350-nucleotide intervening sequence on it*.* The pathogenicity locus also contains *tcdE*, *tcdD,* and *tcdC*.* tcdC is located downstream of tcdA and acts* as a negative regulator of toxin production. However, *tcdD* is *located *upstream of *tcdB* and *acts* as a positive regulator. Also, the gene encoding TcdE is *located *between *tcdB* and *tcdA*) (37). The toxin binds to the target cell receptors via a C-terminal sequence. The toxin is composed of glucosyltransferases (enzymatic domain) and translocates into the cytosol in low-pH conditions. Then, it is activated by inositol hexakisphosphate, and the autoprotease cleaves and releases the glucosyltransferase domain and inactivates Rho, Rac, and Cdc42 in target cells, disturbing the structure of the cytoskeleton and leading to apoptosis and inflammation. Another toxin, CDT is a binary actin-ADP-ribosylating toxin that causes depolymerization of actin (37) and includes CDTa (the enzymatic domain) and CDTb (the binding/translocation domain), and its encoding gene is located on the chromosome. CDTa has ADP ribosyltransferase activity, and CDTb has transfer activity (38). Other virulence factors of *C. difficile* include endospore, S-layers, cell surface polysaccharides (PSI, PSII, and PSIII), fibronectin-binding proteins, flagella, fimbriae, and the heat shock protein GroEL (39). Endospores can persist in inappropriate environments (despite antibiotic treatment) for a long time. Other factors are the cause of attachment and colonization of infection (40). 

***Available vaccines for human clostridial diseases***

There are several vaccines in different types for the mentioned clostridial diseases**, **which are in experimental progress, development, or commercially available in the world. Some of them are presented in Table 1, 2, and 3. 

***Toxoid vaccines***

A purified monovalent *C. botulinum* type F toxoid has been manufactured by inactivation using formaldehyde and adding alum as an adjuvant (41). This toxoid has been surveyed as an intramuscular and subcutaneous vaccine in humans (42). Later, polyvalent (ABEF) toxoid vaccines were developed. This toxoid vaccine is currently administrated to immunize high-risk humans in Japan (43). An improved bivalent (AB) toxoid vaccine was developed and has been administrated in laboratory animals. Later, a trivalent (ABE) toxoid vaccine was prepared (44). Also, an experimental pentavalent (ABCDE) toxoid was developed separately. After purification and chemical inactivation using formaldehyde, adjuvant was added and it was allowed to be used for a limited time due to declining efficacy (44). A commercial bivalent (BoNT/C–D) toxoid vaccine has been also prepared and administrated in domestic animals, especially in cattle (45) and a monovalent (B) toxoid vaccine is being administrated in horse herds (46).

Chemically inactivated toxoids have been developed for immunization of humans. An experimental tetravalent (A, B, E, and F) toxoid vaccine has been engineered and administrated in humans. The results showed no side effects (43). Also, BoNT/A toxoid and a mutated cholera toxin have been prepared and surveyed in mice. The results showed specific antibody production against BoNT (47)(Table 1).

An effective tetanus toxoid vaccine has been developed by Ramon and collaborators in 1926 (48). The vaccine consisted of growing C. tetani in a medium containing glucose, vitamins, inorganic salts, and casein digest. Then, the toxin was inactivated by formaldehyde to turn into toxoid. The toxoid can be purified by ammonium sulfate precipitation (49), ultrafiltration (50), flow filtration (51), and chromatography (52)(Table 2).

Toxoid vaccines have been described for *C. difficile* for over three decades. For the first time, purified toxin A was prepared and analyzed. The results showed that it induced the immune system when administered intra-gastrically (53). Later, purified toxoid-B vaccine adjuvanted with MlipidA/RIBI was developed and administrated to hamsters via the intraperitoneal route. This toxoid vaccine could protect vaccinated animals from mortality (54). In spite of good results obtained in animal models, the use of toxoid-based vaccines in humans has been limited for a long time. Kim and coworkers have developed a bivalent (AB) toxoid vaccine and administrated it in infant hamsters (55). Later, a partially purified toxoid-A and -B vaccine was introduced as the first vaccine candidate in a clinical study for human consumption (56). Ghose and coworkers have prepared another toxoid vaccine based on a cholera toxin (CT) adjuvant for induction of systemic and mucosal immune responses (57). Recently, a highly purified toxoid-A and -B vaccine adjuvanted with aluminum hydroxide has been developed (58, 59). The preclinical results in laboratory animals showed good protection levels against *C. difficile *(58). Also, this toxoid-based vaccine was assessed in a phase I clinical trial in healthy humans. The results showed a good safety level; however, the protection against TcdB toxoid declined six months after vaccination unlike TcdA toxoid (59). Furthermore, the vaccine was evaluated in a phase II trial in humans at risk (60). Then, a recombinant toxoid-based vaccine adjuvanted with AlPO_4_ was developed that consisted of genetically modified TcdA and B toxins (61) and was administrated in hamsters to estimate the rate of protection against the disease. The results showed that the vaccine was nearly effective against oral challenges (Table 3).

Most of the studies on toxoid vaccines have shown that the need for addition of adjuvants exists due to the low molecular weight of the toxoids. However, pure toxoids are recommended because of having no bacteria and no possibility of conversion to virulence. Also, toxoids have lower susceptibility to environmental conditions and are more stable compared with bacterin vaccines (1).

***Recombinant vaccines***

Protein-based vaccines include native, toxoid, and recombinant-engineered vaccines. The previous study have cloned and expressed the HC c-terminal of the BoNT serotype A in *Escherichia coli* and shown that the immune response against HC/A was efficient (62). Also, recombinant HC has been expressed in the yeast *Pichia pastoris* (*P. pastoris*) as a suitable host (63)*.* Furthermore, neurotoxin-associated protein HA-33/A from *C. botulinum *has been expressed in *E. coli* and evaluated in mice. The results showed a high antibody titer in vaccinated mice (64). In another study, the full length of the heavy chain of BoNT/ A has been transformed into* E. coli *(65). These studies have been extended towards developing the BoNT HC vaccine from other serotypes. The BoNT recombinant protein of serotype E has been expressed in *E. coli*. Mice have been vaccinated with purified recombinant protein mixed with Freund’s complete adjuvant at the first step. Subsequently, mice were vaccinated with incomplete adjuvant at the second and third steps of vaccination, and finally with phosphate-buffered saline at the fourth step. The ELISA results showed that the vaccine could protect mice against botulinum neurotoxins after 14 days (66). A variable domain of HC/BoNT serotype E has been cloned and expressed in *P. pastoris*. Based on this result, its expression was higher compared with expression on *E. coli *(67). Researchers cloned and expressed all seven serotypes of botulinum toxin in *P. pastoris,* successfully (68). Later, HC C-terminal BoNT serotypes A and B were fused in *E. coli* (69). A recombinant chimeric vaccine consisting of C-terminal HC of BoNTs serotypes A, B, and E has been developed and evaluated by *in vivo* and *in vitro* assays. The results demonstrated that vaccinated mice were protected against BoNTs serotypes A and E more than against BoNTs serotype B (70). Also, HC BoNT serotypes C and D have been combined, and expressed in* E. coli *and have shown that the recombinant H chains can be used as an effective and safe vaccine in domestic animals (71). In another research, a recombinant chimera vaccine consisting of the LTB (heat-labile enterotoxin B) subunit of *E. coli* fused to the HC of BoNT serotypes C and D and mixed with aluminum hydrochloride was evaluated in mice and guinea pigs. Based on the results, vaccination induced a high immune response. So, this vaccine appears to be a suitable vaccine for the prevention of disease in animals (72). Another recombinant vaccine consisting of the LC of BoNT serotype A was developed and expressed in *E. coli. *Results showed high expression of the light chain of the toxin (73). 

Another approach for developing recombinant vaccines against the BoNT serotype C was to introduce mutations in the light chain by altering the amino acid residues. Then, the mutated gene was expressed in *E. coli*. The results showed that by both subcutaneous and oral administrations to mice, the expressed mutated gene was able to protect against BoNT/C (74). In another research, a mutant vaccine against the BoNT serotype A1 carrying a mutation in the catalytic domain has been developed in *P. pastoris *(75). Also, another vaccine with a single mutation in HC has been developed to lack ganglioside binding to the neurons. This genetically-engineered vaccine has the ability to stimulate protective immunity in mice (76).

Furthermore, LHN/A (including catalytic LC and the Hn translocation domain of BoNT) was mentioned as a BoNT bivalent recombinant vaccine that was effective against botulinum neurotoxin types A or B (77). Multiple amino acid mutations have been also studied to reduce the catalytic potential of the LC of *C. botulinum* neurotoxin as a recombinant vaccine (78)(Table 1).

Development of nanovaccines has been another approach focused on using chitosan nanoparticles containing BoNT serotype E, which have the ability to induce an immune response by oral route in mice (79).

The tetanus toxin gene *tent* was cloned in *E. coli* for the first time (80). Then, the recombinant attenuated Salmonella typhimurium (*S. *typhimurium) carrying the *tent *gene was used for cattle immunization by oral, nasal, and subcutaneous routes. The results showed that the subcutaneous inoculation evoked local and systemic immune responses (81). While oral and nasal inoculation did not evoke systemic immune response against tetC. After that, the C-fragment of the heavy chain was cloned and expressed in *E. coli* as a suitable vaccine candidate (82). Different researchers have been working on recombinant vaccines, which showed the subunit vaccine had good protective ability in laboratory animals (83). Cytomegalovirus has been also used as the carrier of the gene encoding the C-fragment of tetanus heavy chain and evaluated in mice. The results showed the ability of the vaccine to induce the immune system. So, this vaccine has been proposed for use in developing countries (84)(Table 2).

Pilot studies have been done for identification of protective epitopes of *C. difficile* toxins, including *TcdA* and *TcdB* sequences in order to produce recombinant toxin-based peptides (85). For the first time, the recombinant TcdA vaccine was prepared and administrated subcutaneously in hamsters and was protective against death and diarrhea (86). Ryan has developed a recombinant vaccine containing receptor binding domain (RBD) of *C. difficile* toxin A fused to a secretion signal of *E. coli* hemolysin and cloned it into an attenuated *Vibria cholerae* vector. Then, the vaccine was administered orally to rabbits. The results showed this vaccine could induce the immune system against *C. difficile* toxin in the gastrointestinal tract (87)*. *In another study, a recombinant fusion protein containing RBD of TcdA and the fragment C of tetanus toxin was cloned in an attenuated *S. typhimurium* vector and administrated in intragastric and intranasal routes in mice resulting in protective immunity in intestinal and pulmonary mucosa (88). In a similar experience, this recombinant domain was purified and combined with the adjuvanted heat-labile *E. coli* enterotoxin (LT) and mutant of heat-labile *E. coli* enterotoxin (LTR72) and administrated intranasally. The results demonstrated that the recombinant-based vector vaccine induced protective immunity in the pulmonary but not the intestinal mucosa (89). Also, recombinant *B. subtilis* spores were used as vector containing toxins A and B peptide repeats and evaluated as a recombinant vaccine. Based on the obtained results, mice vaccinated with recombinant spores were protected from reinfection (90). Further research has shown that the RBD subdomains of TcdA and the fragment C of tetanus toxin induced anti-TcdA IgG serum response as well as a fecal IgA response in mice. Also, vaccinated hamsters were protected from death following challenge (91). Moreover, a recombinant vaccine containing a C-terminal peptide of toxin A lacking ADP-ribosyltransferase activity was cloned in *E. coli* (92). A DNA vaccine containing RBD of TcdA has been developed and generated protective immunity against TcdA toxin in mice (93). Also, an adenovirus-based vaccination against RBD of TcdA showed sufficient humoral and cellular immune response in mice (94). To increase the efficacy, Tian and colleagues have developed a fusion protein containing RBDs of TcdA and TcdB, which was administrated in laboratory animals intramuscularly. The results showed this fusion protein generated antibody response against both toxins in mice; although antibody production against TcdA was protective even without adjuvant, antibody titer against TcdB was not enough for protection. Also, the vaccination of hamsters with the adjuvanted vaccine was protective. Then, the fusion protein with alum hydroxide, as an adjuvant, was tested in a non-human primate model. The fusion protein was tested in a clinical trial (phase I) and induced antibodies against TcdA and B (95). The RBDs of TcdA and TcdB + flagellin of* S. typhimurium were used for *stimulation of the immune system in mice. Based on the results, the recombinant toxin-based peptides vaccine adjuvanted with *S. typhimurium* flagellin could protect laboratory animals following challenge (96). On the other hand, flagellin from *C. difficile* has been measured by ELISA in the culture of cell lines and was proved as an effective vaccine in the occurrence of *C. difficile* disease (97)(Table 3). 

Recombinant technology is a non-toxic, high-throughput, and promising tool for vaccine development; however, it is not a fully advantageous process. Production of insoluble recombinant proteins is time-consuming and difficult with multiple stages of solubilization and refolding. Bacterial remains such as lipopolysaccharides (LPS), present in the recombinant toxoid purifications, are another uncertainty about the worldwide use of these vaccines (1). Also, the need for addition of adjuvants still exists.

***Nucleic acid-based vaccines***

Plasmid- and viral-based vectors are being developed against BoNT. Clayton and Middlebrook have used the HC encoding gene of BoNT serotype A as a useful method of vaccination (98). In another effort, a DNA vaccine consisting of the gene encoding fragment C of BoNT serotype A, which contained a Cytomegalovirus (CMV) derived promoter was used for immunization against botulinum toxin in mice (99). Also, DNA based vaccine against botulinum neurotoxin serotypes A, B, and E has been prepared and delivered using DNA electrotransfer leading to a high level of neutralizing antiserum titers against botulinum toxin in mice (100). Furthermore, DNA vaccination against botulinum type F resulted in high levels of antibody in mice (101). Also, monovalent and trivalent DNA vaccines with HC C-terminal BoNT serotypes A, B, and E have been developed which induced a specific immune response in mice (102).

 Several viral-based vectors have been developed for immunization against the botulinum toxin. Adenovirus-based vectors containing a C-fragment heavy chain of BoNT/C have been evaluated as a candidate oral vaccine in mice (103)*. *Li *et al*. have administrated a live attenuated influenza virus as a virus-based vector for vaccination against BoNT/A (104)*. *Hudacek has developed a recombinant RABV (recombinant rabies virus) to make a candidate trivalent vaccine against BoNT/A, BoNT/B, and BoNT/C. Vaccination with the trivalent vaccine has shown effective protection against BoNT/A and BoNT/B, but not BoNT/E (105).

Yu *et al*. have developed Semliki Forest Virus-based viral vectors containing HC/A, HC/B, HC/E, HC/F, and HC/TeNT (tetanus neurotoxin) and used them for protection against challenge. The results showed this vaccine could protect against the heavy chain of BoNT serotype and neutralize the tetanus neurotoxin in mice (106). A Venezuelan equine encephalitis virus-based vector containing HC/A has been prepared and used for vaccination of mice and the results showed mice survival after challenge (107)(Table 1).

Genetically-engineered vaccines are forthcoming favorable alternatives for traditional vaccines, because of decrease in several stages of bacterial culture and vaccine production, and easy production. However, there are unfavorable disadvantages, which mask the above-mentioned benefits. In this regard, the risk of integration of vaccine DNA in the host genome, low immunogenicity and need for boosters or adjuvants, and sometimes instability can be mentioned (1).

***Surface-associated antigens***

Another group of vaccines is the surface-associated proteins and polysaccharides; since the antibodies raised against these antigens can decrease *C. difficile* infection, although the protection is not as sufficient as toxoid or genetically engineered vaccines (108). Antibody production against flagellar components such as FliC and FliD, Cwp66 adhesin, fibronectin-binding protein Fbp68, cysteine protease Cwp84 and s-layer proteins in combination with adjuvants, in patients with *C. difficile* infection have proved to be effective for immunization (109). In one case, the *C. difficile* protease Cwp84 was prepared as a recombinant antigen and formulated with Freund’s adjuvant, cholera toxin and without adjuvant and the immune response in hamsters induced by subcutaneous, rectal, and intragastric was evaluated, respectively. The results showed a significant decrease in colonization and greater survival compared with the control group (110). In another experiment, the* C. difficile* Cwp84 was encapsulated in pectin beads and administrated by the oral route in hamsters. Nearly half of the vaccinated hamsters survived longer than the control group after the challenge (111). Also, polysaccharides on the surface of *C. difficile*, named PSI, PSII, and PSIII, especially PSIII have been found efficient for immunization (112-114). The PSII polysaccharide has been conjugated to diphtheria toxoid CRM197, mixed with MF59 adjuvant, and administrated in BALB/C mice resulting in high levels of IgG (112). Also, the PSII polysaccharide conjugated to LTB of *E. coli* induced the immunogenic response in rabbits (113). The PSIII conjugated to the inactivated *Pseudomonas aeruginosa* (*P. aeruginosa*) ExoA *P. aeruginosa*/HSA (human serum albumin) and mixed with Freund’s adjuvant has been administrated intraperitoneally or subcutaneously in BALB/C mice and rabbits. The results demonstrated the production of IgG antibodies in animals (114). These researches introduce surface-associated antigens as suitable targets for vaccine development against *C. difficile *infection (Table 3). 

**Table 1:** Trial and commercial vaccines against* Clostridium botulinum*

**Material**	**Vaccine**		**Strain**	**Ref**	**Comment**
**Protein**	**toxoid**		***C. botulinum*** ** type** ** A**	**(** 47 **)**	**Need adjuvant, more stable than bacterin vaccines**
			***C. botulinum*** ** type** ** B**	**(** 46 **)**	
			***C. botulinum*** ** type F**	**(** 41 **)**	
			**bivalent (AB)**	**(** 44 **)**	
			**bivalent (CD)**	**(** 45 **)**	
			**trivalent (ABE)**	**(** 44 **)**	
			**polyvalent (** **ABEF)**	**(** 43 **)**	
			**pentavalent (ABCDE)**	**(** 44 **)**	
	**Recombinant HC**		**BoNT serotype A**	**(** 62 **, ** 63 **)**	**Non-toxic, high-throughput** **,** ** and promising tool for vaccine development, time-consuming and difficult with multiple stages of solubilization and refolding, ** **Need adjuvant**
			**BoNT ** **serotype E**	**(** 66 **, ** 67 **)**	
			**bivalent (AB)**	**(**69**)**	
			**bivalent (CD)**	**(** 71 **)**	
			**trivalent ** **(ABE)**	**(** 70 **)**	
	**Recombinant ** **LC**		**BoNT serotype A**	**(** 73 **)**	
			**BoNT serotype C**	**(** 74 **)**	
	**Recombinant LHN (** **Light chain plus translocation domain)**		**bivalent (AB)**	**(** 77 **)**	
**Nucleic acid**	**Plasmid (HC** **-** **based vaccine)**		**BoNT** ** serotype A**	**(** 98 **)**	**easy production,** **low immunogenicity and need booster or adjuvants**
	**Virus**	**Adenovirus-based vectors**	**BoNT/C**	**(** 103 **)**	
		**influenza virus**	**BoNT/A**	**(** 104 **)**	
		**Rabies**	**trivalent ** **(ABC)**	**(** 105 **)**	
		**Semliki Forest**	**HC/ABEF HC/TeNT**	**(** 106 **)**	
		**Venezuelan equine encephalitis**	**HC/A**	**(** 107 **)**	

**Table 2:** Vaccines produced against *Clostridium tetani* in the world

**material**	**Vaccine**		**Strain**	**Ref**	
**Protein**	**toxoid**		***C. tetani***	**(** 48 **)**	
**Nucleic acid**	**Bacteria**	***Salmonella typhimurium***	***C. tetani***	**(** 81 **)**	
		***E.*** ***coli *(HC)**		**(** 82 **)**	
	**Virus**	**Cytomegalovirus (HC)**		**(** 84 **)**	
				


**Table 3:** Trial and commercial vaccines against *Clostridium difficile*

**Material**	**Vaccine**	**Antigens**	**Adjuvant**	**Route of immunization**	**Animal model**	**Ref**
**Protein**	**Toxoid**	**Purified toxoid A**	**-**	**intra-gastrically**	**-**	**(** 53 **)**
		**purified toxoid-B**	**MlipidA/RIBI**	**intraperitoneal**	**Hamster**	**(** 54 **)**
		**Toxoids** ** A and B**	**Freund**	**subcutaneous**	**Hamster**	**(** 55 **)**
		**Highly purified ** **toxoids** ** A and B**	**Al(OH)**_3_	**intramuscular**	**Hamster**	**(** 58 **)**
		**Genetically modified ** **toxoids** ** A and B**	**AlPO**_4_	**intramuscular**	**Hamster**	**(** 61 **)**
	**Recombinant**	**RBD TcdA**	**Freund**	**subcutaneous**	**Hamster**	**(** 86 **)**
		**RBD TcdA**	**Cholera toxin**	**Oral**	**Rabbit**	**(** 87 **)**
		**RBD TcdA**	**-**	**Intranasal, intragastric**	**Mice**	**(** 88 **)**
		**RBD TcdA/B**	**-**	**Oral,**	**Mice, hamster**	**(** 90 **)**
		**RBD, CP, TcdA+B**	**Al(OH)**_3/_ **MF59**	**intraperitoneal**	**Mice, hamster**	**(** 91 **)**
		**ToxinA/B chimeric protein**	**-**	**Intramuscular,** **intraperitoneal**	**Mice, hamster**	
		**RBD TcdA+B**	**FliC /None/ Al(OH)**_3/_ **LT192**	**Intranasal**	**Mice**	**(** 96 **)**
		**Fusion protein RBD TcdA+B**	**None/Al(OH)**_3_	**Intramuscular**	**Mice, hamster, monkey**	**(** 95 **)**
		**RBD TcdA- peptide**	**-**	**Intramuscular**	**Mice**	**(** 93 **)**
		**RBD TcdA- peptide**	**-**	**Intramuscular**	**Mice**	**(** 94 **)**
**Surface proteins**		**Crude SLP**	**Al(OH)**_3_ **/ CT /RIBI/chitosan glutamate/TMC**	**Intranasal, intraperitoneal**	**Mice, hamster**	**(** 109 **)**
		**Cwp84**	**-**	**intra-gastrically**	**Hamster**	**(** 111 **)**
		**Cwp84**	**None/ Freund/CT**	**Subcutaneous, rectal, intra-gastrically**	**Hamster**	**(** 110 **)**

## Conclusion

Clostridial diseases have been recognized in humans and animals since centuries ago. Foodborne botulism especially in infants has caused great economic losses. Botulism has been first identified in 1895; however, the first record goes back to 1735. Since then, clostridial toxins have been discovered and several diagnostic and preventive methods have been developed. Many clostridial vaccines are now commercially available all over the world. However, some commercial vaccines are not available worldwide, especially in most developing countries. Traditional toxoid vaccines, although offering proper immunity, need inactivation steps, have a time-consuming production process, and the issue of residual formaldehyde remains. Hence, there is always a need for new-generation vaccines. Also, new vaccine alternatives may have a greater impact on faster eradication of human clostridial diseases. Several experimental or commercial vaccines have been developed in recent years. Recent advances in increasing immunogenicity have made the new-generation vaccines more popular. Studies on different inoculation strategies have implicated the mucosal immunization or intranasal route results in effective immunity in some vaccines and can be a good alternative for other inoculation routes. Formulations with appropriate adjuvants have been studied for optimum stimulation of the immune system. Most of the new-generation vaccines such as recombinant toxin proteins are produced at experimental levels and need more approval steps to be widely used as human vaccines. The most important challenge in the production of a desirable vaccine is its ability to stimulate the immune response in the host. New vaccine strategies such as DNA vaccines, and recombinant and toxoid vaccines need more focus on the multiple steps leading to their production, such as antigen presentation, recombinant protein concentration, adjuvant requirements, detoxification, and route of administration. Gathering the desirable aspects of different types of human clostridial vaccines in this review showed their promising application in the vaccination programs, especially in the current epidemiologically changing environment. 

## Authors’ Contributions

LAK had the idea for the article, performed the literature search and prepared the original draft. AZ revised the manuscript.

## Conflicts of Interest

The authors declare no competing interests.
